# Sexually dimorphic gene expression that overlaps maturation of type II pneumonocytes in fetal mouse lungs

**DOI:** 10.1186/1477-7827-4-25

**Published:** 2006-05-04

**Authors:** Marc Simard, Pierre R Provost, Yves Tremblay

**Affiliations:** 1 Laboratory of Ontogeny and Reproduction, CHUQ, PCHUL, Faculty of Medicine, Laval University, Québec City, Québec, Canada; 2 Ob/Gyn Department, Faculty of Medicine, Laval University, Québec City, Québec, Canada; 3 Centre de Recherche en Biologie de la Reproduction (CRBR), Laval University, Québec City, Québec, Canada

## Abstract

**Background:**

In human, respiratory distress of the neonates, which occurs in prematurity, is prevalent in male. Late in gestation, maturation of type II pneumonocytes, and consequently the surge of surfactant synthesis are delayed in male fetuses compared with female fetuses. Although the presence of higher levels of androgens in male fetuses is thought to explain this sex difference, the identity of genes involved in lung maturation that are differentially modulated according to fetal sex is unknown. We have studied the sex difference in developing mouse lung by gene profiling during a three-day gestational window preceding and including the emergence of mature PTII cells (the surge of surfactant synthesis in the mouse occurs on GD 17.5).

**Methods:**

Total RNA was extracted from lungs of male and female fetal mice (gestation days 15.5, 16.5, and 17.5), converted to cRNA, labeled with biotin, and hybridized to oligonucleotide microarrays (Affymetrix MOE430A). Analysis of data was performed using MAS5.0, LFCM and Genesis softwares.

**Results:**

Many genes involved in lung maturation were expressed with no sex difference. Of the approximative 14 000 transcripts covered by the arrays, only 83 genes presented a sex difference at one or more time points between GDs 15.5 and 17.5. They include genes involved in hormone metabolism and regulation (i.e. steroidogenesis pathways), apoptosis, signal transduction, transcriptional regulation, and lipid metabolism with four apolipoprotein genes. Genes involved in immune functions and other metabolisms also displayed a sex difference.

**Conclusion:**

Among these sexually dimorphic genes, some may be candidates for a role in lung maturation. Indeed, on GD 17.5, the sex difference in surfactant lipids correlates with the sex difference in pulmonary expression of apolipoprotein genes, which are involved in lipid transport. This suggests a role for these genes in the surge of surfactant synthesis. Our results would help to identify novel genes involved in the physiopathology of the respiratory distress of the neonates.

## Background

Hyaline membrane disease (respiratory distress of the neonate) occurs primarily in premature infants. A major cause of this disease is surfactant deficiency. Hyaline membrane disease and surfactant synthesis are both affected by fetal sex. Indeed, the surge of surfactant synthesis is normally delayed in the developing male lung when compared with the female, while hyaline membrane disease is prevalent in males [1-5].

Surfactant synthesis occurs in type II pneumonocytes (PTII) following maturation of these cells, which is promoted by fibroblast-PTII cell communication [5]. This maturation process is stimulated by glucocorticoids [6] and involves some cytokines including epidermal growth factor (EGF) [7,8], neuregulin-1 [9], and keratinocyte growth factor (KGF) [10] as positive regulators and transforming growth factor-β1 (TGF-β1) [11-14] as negative regulator.

Androgens have been shown to both delay fetal lung maturation *in vivo *[3,15] and block the stimulatory effect of corticosteroids on surfactant synthesis *in vitro *[16]. These effects are mediated through binding of androgens to androgen receptors [3,15,17], which are present in both male and female lung tissues [18,19]. There is an active androgen metabolism in the developing lung where androgen synthesis [18,20] and inactivation [18,21] occur. In mice, many genes involved in androgen metabolism are regulated specifically on gestation day (GD) 17.5 in parallel with the emergence of mature PTII cells ([18] and unpublished data).

The surge of surfactant synthesis occurs on GD 17.5 in the mouse [3,18] with a sex difference in pulmonary surfactant lipid levels [3]. Male mice carrying the *Tfm *gene (male with testicular feminization), which have no functional androgen receptors, have surfactant levels comparable with those of normal females at a comparable developmental time point [3]. Therefore, the mouse is a good model to study the effect of fetal sex on the timing of the developmental events related to the surge of surfactant synthesis.

Knowing that the surge of surfactant synthesis is delayed in male fetal mouse lungs compared with females, we were interested to identify genes that are expressed with a sex difference during the gestational period that overlaps the surge of surfactant production. We found, by microarray analysis, genes exhibiting a sex difference in expression in lung development during a three-day window preceding and including the emergence of mature PTII cells. To date, no gene profiling study of sex differences in the embryonic lung tissue exists. Using Affymetrix technology, we have studied about 14,000 transcripts and variants with more than 22,600 probe sets in male and female fetal lungs on GDs 15.5, 16.5, and 17.5.

## Methods

### Animals

Protocols were approved by the animal care and use committee and the institutional review board of the Centre de Recherche du Centre Hospitalier Universitaire de Québec (protocol 2002-080). Balb/C mice (*Mus musculus*) were mated during the night. Appearance of the copulatory plug was considered as gestation day 0.5 (GD 0.5). Pregnant females were euthanized by exposure to CO_2_. Fetal sex was determined by examination of the genital tract with a dissecting microscope at 15 × magnification. Fetal lungs were collected and one pool of tissues was prepared for each sex and each pregnant animal prior RNA extraction.

### RNA extraction

Total RNA was extracted using Tri-reagent, a mixture of phenol and guanidine thiocyanate in a monophasic solution (Molecular Research Center, Cincinnati, OH) as described previously [22]. Each RNA sample was purified on a CsCl gradient as described [23], using a TLA 120.2 rotor in an Optima MAX centrifuge (Beckman, Mississauga, ON, Canada).

### Preparation of probes

Samples were processed following the Small Sample Labeling Protocol version II from Affymetrix [24]. This protocol is based on the principle of performing two cycles of cDNA synthesis and *in vitro *transcription reactions for target amplification. Briefly, 10 μg of total RNA were converted to cDNA by incubation with 400 units of SuperScript II reverse transcriptase (Invitrogen, Carlsbad, CA), a T7 oligonucleotide-d(T)_24 _as a primer (5'-GGCCAGTGAATTGTAATACGACTCACTATAGGGAGGCGG(T)_24_-3'), combined with 1 mM dNTPs (deoxynucleotide triphosphates) in 1 × first strand buffer (50 mM Tris HCl pH 8.3, 75 mM KCl, 3 mM MgCl_2_, 10 mM DTT) at 42°C for 1 h. Second strand cDNA synthesis was performed using 40 units of DNA polymerase I (Invitrogen), 10 units of *E. coli *DNA ligase (Invitrogen), 2 units of RNase H (Invitrogen), and 0.2 mM dNTPs in 1 × reaction buffer (18.8 mM Tris-HCl pH 8.3, 90.6 mM KCl, 4.6 mM MgCl_2_, 3.8 mM DTT, 0.15 mM NAD, 10 mM (NH_4_)_2_SO_4_) at 16°C for 2 h. Each cDNA sample was blunt ended by addition of 10 units of T4 polynucleotide kinase (Invitrogen) and incubation at 16°C for 10 min. cDNA samples were purified by phenol-chloroform extraction using phase lock gels (Brinkmann Instruments Inc., Mississauga, ON, Canada), ethanol precipitated and resuspended in 10 μl of DEPC- (diethylpyrocarbonate) treated H_2_O. First cycle amplification was performed using a MEGAscript T7 Kit (Ambion, Austin TX). The mixture (10 μl final volume) was incubated at 37°C for 5 h. cRNA was purified using a RNeasy Mini Kit (Qiagen, Valencia, CA) according to the protocol of the manufacturer. Purified cRNA was reverse-transcribed to cDNA for a second time following the protocol used for the first cycle. For the second amplification, a T7 BioArray High Yield RNA Transcript Labeling Kit (Enzo Diagnostics, Farmingdale, NY) was used to produce biotinylated cRNA. The mixture (20 μl final volume) was incubated at 37°C for 5 h with gentle mixing every 30 min. Labelled cRNA was purified using a RNeasy Mini Kit (Qiagen) according to the protocol of the manufacturer. Purified cRNA was fragmented into segments of 20–300 nucleotide length by incubation in a fragmentation buffer (100 mM potassium acetate, 30 mM magnesium acetate, 40 mM Tris-acetate pH 8.1) for 20 min at 94°C. The quality of cRNA amplification and cRNA fragmentation was monitored by micro-capillary electrophoresis (Bioanalyser 2100, Agilent Technologies, Mississauga, ON, Canada).

### Microarray hybridization, scanning, and analysis

Each preparation of cRNA probe was hybridized to two GeneChip Mouse Genome 430 A arrays (Affymetrix, Santa Clara, CA). Each microarray was pre-hybridized in 1 × hybridization buffer (0.1 mg/ml herring sperm DNA, 0.5 mg/ml acetylated BSA) at 45°C for 10 min under constant rotation (60 rpm). Then, the buffer was replaced by a mixture containing 15 μg of fragmented cRNA in 1 × hybridization buffer, and the following internal controls from Affymetrix: 5 nM control oligonucleotide B2 and 1 × eukaryotic hybridization control (1.5 pM *BioB*, 5 pM *BioC*, 25 pM *BioD *and 100 pM *cre*). Samples were incubated at 45°C for 16 h under constant rotation. Microarrays were processed using an Affymetrix GeneChip Fluidic Station 400 (protocol EukGE-WS2Av4). Staining was initiated with streptavidin-conjugated phycoerythrin (SAPE), followed by amplification using a biotinylated anti-streptavidin antibody and by a second round of SAPE. GeneChips were scanned using a GeneChip Scanner 3000 with autoloader (Affymetrix). Data acquisition and analysis were performed using the Microarray Suite 5.0 software (Affymetrix). Signal intensities for β-actin and GAPDH genes were used as internal quality controls; their ratio of fluorescence intensities for the 5' and 3' ends was <2. Differentially expressed genes were determined using the LFCM software [25]. Briefly, variable fold change limit (LFC) decreasing with gene expression value was used to select differentially expressed genes. The LFC equation is Y = a + b/X, were X is the minimum gene expression intensity from two arrays and Y is the fold change limit. The parameters a and b were estimated from the distribution of ratios calculated from replicated chips. The resulting cut-off point, Y = 1.8 + 62.0/X, gave an approximately constant rate of false positive modulated genes of 0.1%. All the genes having a fold change above this curve were considered significantly modulated. Data were also analysed with the Genesis 1.6.0 Beta1 software [26]. The MOE430A microarray provides coverage of over 22,600 probe sets corresponding to about 14,000 transcripts and variants. The probe sets were selected from sequences derived from GenBank, dbEST and RefSeq. The sequence clusters were created from the UniGene database (Build 107, June 2002) and then refined by analysis and comparison with the publicly available draft assembly of the mouse genome from the Whitehead Institute Center for Genome Research (MSCG, April 2002) (for more details see [24]).

## Results

Pregnant Balb/C mice were sacrificed on GDs 15.5, 16.5, and 17.5. Total RNA prepared from male and female fetal lungs, from each litter, was pooled in order to obtain the following six samples: GD 15.5 males; GD 15.5 females (each from 2 litters); GD 16.5 males; GD 16.5 females (each from 2 litters); GD 17.5 males; and GD 17.5 females (each from 1 litter) (number of fetuses per RNA preparation were respectively of 6, 9, 7, 9, 2, and 4). Previously, the litters corresponding to GDs 15.5 and 17.5 have shown sex differences in expression for several genes [18,27]. Microarray analysis was performed using six probes prepared from these RNA samples and 12 GeneChip Mouse Genome 430 A array (in duplicate). Values obtained for each gene were compared between sexes for each GD.

### Validation of data

Internal controls assessing the validity and reproducibility of the data were satisfied (see Methods). For each experiment, Table 1 shows the number of probe sets detected (*p*-value = 0.05 based on raw signals obtained for each probe set). Of these, the number of associated known and unknown transcripts was determined from analysis of probe set IDs using NetAffx (Affymetrix) and Excel (Microsoft) softwares. Expressed sequence tags (ESTs), Riken sequences, and all other unknown transcripts ("hypothetical","similar to"...) were sorted into the category named "Unknowns".

**Table 1:** Overview of the microarray data characteristics

	**GD15.5**		**GD16.5**		**GD17.5**	
	**M**	**F**	**M**	**F**	**M**	**F**

Detected probesets (% present)*	12955 (57,3%)	12988 (57,4%)	12660 (56%)	12455 (55%)	12234 (54,1%)	12808 (56,6%)
**Known genes**^†^	6490	6464	6466	6424	6321	6561
**Unknown transcripts**^†^	2406	2433	2030	2019	1965	2060

*- Number of probe sets detected (p-value ≤ 0.05) relative to the total number of probe sets on the array

^†^- When multiple probe sets are associated to the same transcript, only one is considered M, male; F, female

Sex determination of fetuses was confirmed by analysis of several genes associated with the Y-chromosome. Specifically, Ddx3y (DEAD (Asp-Glu-Ala-Asp) box polypeptide 3, Y-linked) (Id. #56, Table 2), Jarid1d (jumonji, AT rich interactive domain 1D (Rbp2 like)) (Id. #57), also called Smcy, Eif2s3y (Y chromosome-encoded subunit of the initiation of translation factor Eif2) (Id. #50) and Uty (ubiquitously transcribed tetratricopeptide repeat gene, Y chromosome) (Id. #65) were expressed, as expected, exclusively in male samples for each GD studied (Table 2).

**Table 2:** Differentially expressed genes in the lung according to sex at GDs 15.5, 16.5 and 17.5

Id*	**Accession number**	**Gene name**^†^		**Fold changes**^‡^			
			
			**Symbol**	**GD15.5 (62)**^§^	**GD16.5 (33)**	**GD17.5 (38)**	**Chr.**
**Apoptosis**							
#1	BI662863	Rho-associated coiled-coil forming kinase 1	Rock1		2.25		
#2	NM_011997	caspase 8 associated protein 2	Casp8ap2		2.15		4 11.4 cM
**Cell adhesion**							
#3	NM_009925	procollagen, type X, alpha 1	Col10a1	M			10 22.0 cM
#4	L20232	integrin binding sialoprotein	Ibsp	M			5 56.0 cM
#5	NM_031163	procollagen, type II, alpha 1	Col2a1	2.96	F		15 54.5 cM
#6	NM_007729	procollagen, type XI, alpha 1	Col11a1	2.25			3 53.1 cM
#7	AK004383	procollagen, type IX, alpha 1	Col9a1	1.96			1 15.0 cM
#8	U08020	procollagen, type I, alpha 1	Col1a1			(1.77)	11 56.0 cM
**Cell growth**							
#9	NM_008341	insulin-like growth factor binding protein 1	Igfbp1	M			11 1.3 cM
**Coagulation**							
#10	NM_008877	plasminogen	Plg	F		F	17 7.3 cM
#11	AK011118	fibrinogen, B beta polypeptide	Fgb	F		F	3 48.2 cM
#12	NM_133862	fibrinogen, gamma polypeptide	Fgg	(3.81)		F	3 41.3 cM
#13	BC005467	fibrinogen, alpha polypeptide	Fga	(3.10)		F	3 44.8 cM
#14	NM_010168	coagulation factor II	F2			(2.67)	2 47.5 cM
**Endopeptidase activity**							
#15	BC012874	serine (or cysteine) proteinase inhibitor, clade A, member 1a	Serpina1a	F		F	12 51.0 cM
#16	NM_007443	alpha 1 microglobulin/bikunin	Ambp	F		F	4 30.6 cM
#17	NM_010582	inter-alpha trypsin inhibitor, heavy chain 2	Itih2	F		F	2 1.0 cM
#18	NM_008407	inter-alpha trypsin inhibitor, heavy chain 3	Itih3	F		F	
#29	NM_007618	serine (or cysteine) proteinase inhibitor, clade A, member 6	Serpina6	(2.66)		F	12 51.0 cM
#20	NM_019429	protease, serine, 16 (thymus)	Prss16		(2.65)		13 10.0 cM
**Erythroid-associated**							
#21	NM_013848	erythroblast membrane-associated protein	Ermap	F			4 57.0 cM
#22	AF069311	Rhesus blood group CE and D	Rhced	F			4 65.7 cM
#23	NM_053149	hemogen	Hemgn	(3.29)	F	F	
#24	NM_010635	Kruppel-like factor 1 (erythroid)	Klf1	(3.01)			
#25	NM_133245	erythroid associated factor	Eraf	(2.38)			
#26	NM_010369	glycophorin A	Gypa	(2.27)			8 36.0 cM
#27	AJ007909	erythroid differentiation regulator 1	Erdr1			(1.75)	
**Hormone metabolism/regulation**							
#28	NM_008293	hydroxysteroid dehydrogenase-1, delta<5>-3-beta	Hsd3b1	M			3 49.1 cM
#29	AV021656	aldo-keto reductase family 1, member B7	Akr1b7	M			6 14.0 cM
#30	NM_009995	cytochrome P450, family 21, subfamily a, polypeptide 1	Cyp21a1	M			
#31	AI195150	group specific component	Gc	F		F	5 44.0 cM
#32	BG141874	transthyretin	Ttr	(3.93)	F	F	18 7.0 cM
#33	NM_031192	renin 1 structural	Ren1	3.67	F		
#34	NM_007423	alpha fetoprotein	Afp	(2.64)		F	5 50.0 cM
#35	U63146	retinol binding protein 4, plasma	Rbp4	(1.89)		(2.16)	19 38.0 cM
**Immune functions**							
#36	NM_011558	T-cell receptor gamma, variable 4	Tcrg-V4	F	F	F	
#37	NM_009019	recombination activating gene 1	Rag1		F	F	2 56.0 cM
#38	M58149	CD3 antigen, gamma polypeptide	Cd3g	F	F		9 26.0 cM
#39	AF119253	histocompatibility 2, class II antigen A, alpha	H2-Aa		F		
#40	AV058500	P lysozyme structural	Lzp-s	3.42		1.70	
#41	X67128	T-cell receptor beta, variable 13	Tcrb-V13	(1.97)	F	F	6 20.5 cM
#42	BC011474	lymphocyte protein tyrosine kinase	Lck		(2.87)		4 59.0 cM
#43	BC003476	Ia-associated invariant chain	Ii		(2.10)		18 32.0 cM
**Lipid metabolism**							
#44	NM_013475	apolipoprotein H	Apoh	F		F	11 63.0 cM
#45	NM_009692	apolipoprotein A-I	Apoa1	F		F	9 27.0 cM
#46	NM_009695	apolipoprotein C-II	Apoc2	(3.20)		F	7 4.0 cM
#47	NM_013474	apolipoprotein A-II	Apoa2	(2.27)		F	1 92.6 cM
#48	BC002148	fatty acid binding protein 4, adipocyte	Fabp4	2.33			3 13.9 cM
#49	BG922397	p47 protein	p47	1.84			
**Protein biosynthesis**							
#50	NM_012011	eukaryotic translation initiation factor 2, subunit 3, structural gene Y-linked	Eif2s3y	M	M	M	
#51	NM_009079	ribosomal protein L22	Rpl22	1.86			
#52	AI642440	ribosomal protein S13	Rps13	(1.81)			
**Signal transduction**							
#53	X67702	secretoglobin, family 1A, member 1 (uteroglobin)	Scgb1a1	M			
#54	BF537798	receptor (calcitonin) activity modifying protein 2	Ramp2	2.07	2.16		11 61.5 cM
#55	NM_016721	IQ motif containing GTPase activating protein 1	Iqgap1	1.96			7 39.0 cM
**Transcriptionnal regulation**							
#56	AA210261	DEAD (Asp-Glu-Ala-Asp) box polypeptide 3, Y-linked	Ddx3y	M	M	M	Y 2.07 cM
#57	AF127244	jumonji, AT rich interactive domain 1D (Rbp2 like)	Jarid1d	M	M	M	Y 2.03 cM
#58	AA617392	Max protein	Max		F		12 32.0 cM
#59	BB393998	flap structure specific endonuclease 1	Fen1	3.70	(2.96)		
#60	NM_007496	AT motif binding factor 1	Atbf1			(2.16)	8 E1
**Transport**							
#61	BC024643	albumin 1	Alb1	(2.71)		(8.68)	5 50.0 cM
#62	AF440692	Transferring	Trf	(2.02)		(3.29)	9 56.0 cM
#63	BB448377	solute carrier family 4 (anion exchanger), member 1	Slc4a1	(2.07)			11 62.0 cM
#64	NM_026331	mitochondrial solute carrier protein	Mscp	(1.90)			
**Others**							
#65	BB742957	ubiquitously transcribed tetratricopeptide repeat gene, Y chr.	Uty	M	M	M	Y 2.06 cM
#66	AI256465	alpha-2-HS-glycoprotein	Ahsg	F		F	16 15.0 cM
#67	AK003182	myosin, light polypeptide 1	Myl1		F		1 34.1 cM
#68	BG806300	inactive X specific transcripts	Xist	(3.34)	(13.22)	(2.41)	X 42.0 cM
#69	AV224521	Gelsolin	Gsn	3.27			2 24.5 cM
#70	NM_025711	Asporin	Aspn	2.72			
#71	BC002136	coronin, actin binding protein 1A	Coro1a			(2.48)	7 62.5 cM
#72	AK017440	RNA imprinted and accumulated in nucleus	Rian		(2.27)		12 54.5 cM
#73	AV156640	expressed in non-metastatic cells 1, protein	Nme1	2.23			
#74	AF291655	Tenomodulin	Tnmd	2.12			
#75	BB745314	male-specific lethal-2 homolog (Drosophila)	Msl2		(2.11)		
#76	BB490338	calponin 3, acidic	Cnn3	2.04			
#77	BB475271	LUC7-like 2 (S. cerevisiae)	Luc7l2		1.96		
#78	NM_053123	SWI/SNF related, matrix associated, actin dependent regulator of chromatin, subfamily a, member 1	Smarca1		1.93		
#79	BM207360	unc-50 homolog (C. elegans)	Unc50			1.91	
#80	AV011848	malate dehydrogenase 1, NAD (soluble)	Mdh1	1.89			11 12.0 cM
#81	AA270173	Lamin B1	Lmnb1		(1.89)		18 29.0 cM
#82	BB192717	protein phosphatase 2 (formerly 2A), reg. subunit A (PR 65), alpha isoform	Ppp2r1a		(1.88)		
#83	BE372352	ARP3 actin-related protein 3 homolog (yeast)	Actr3	1.77			
**Unknowns**							
#84	AA717264	Transcribed sequences			2.51		
#85	BC021831	CDNA clone MGC:67258 IMAGE:6413648, complete cds			(2.14)	(2.36)	
#86	AK018316	DNA segment, Chr 2, ERATO Doi 145, expressed	D2Ertd145e		2.31		2 31.0 cM
#87	BE865094	expressed sequence AI448196	AI448196			(2.14)	
#88	BB151477	RIKEN cDNA 2810468K05 gene	2810468K05Rik		1.88		

*- Identification numbers of genes found in this study. ^†^- Genes are presented in only one functional category although some of them could have been placed in more than one category. ^‡^- Values with no bracket correspond to higher levels for males, while values in brackets correspond to higher levels for females. The letter M (Male) or F (Female) replace the fold change value when the gene was expressed in one sex only. ^§^- The number of genes differentially expressed at each gestational day (GD) is indicated.

**Figure 1:**
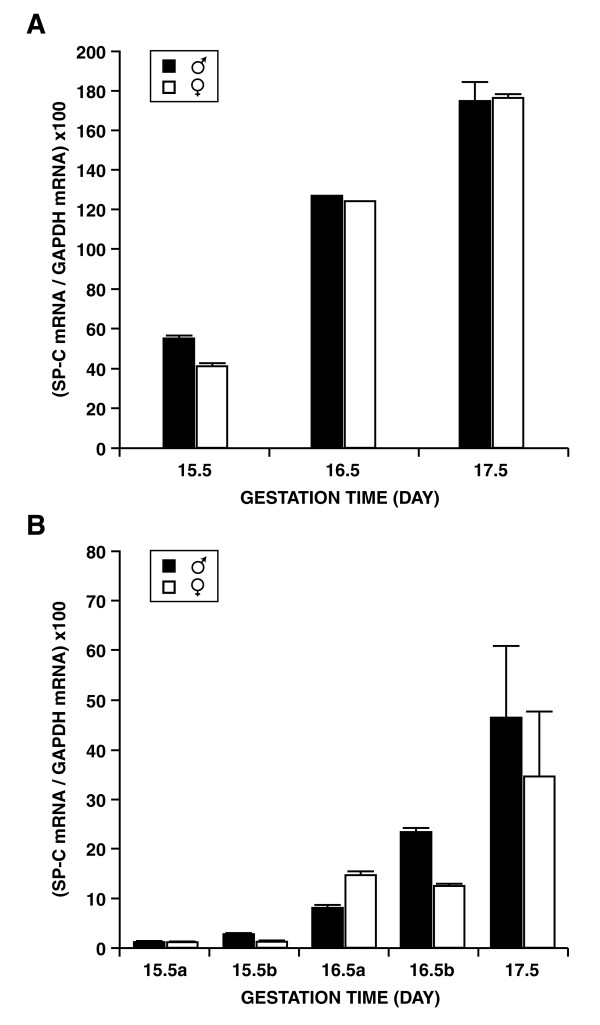
**Relative levels of SP-C gene expression on GDs 15.5, 16.5 and 17.5 **Relative levels of SP-C gene expression (± SD) on GDs 15.5, 16.5 and 17.5 obtained by microarray hybridization (A) and real-time PCR (B). RNAs corresponding to samples "a" and "b" in panel B were pooled prior to preparation of probes used in panel A. "a" and "b" refer to different litters.

The expression patterns of surfactant associated protein C (SP-C) obtained from microarray experiments (Figure 1A) and real time PCR (Figure 1B) using the same RNA preparations are presented. The reported increase in SP-C mRNA was observed in both cases.

### Several genes of interest exhibiting no sex difference in expression

Several genes involved in lung development and expressed with no sex difference in our study are presented in Table 3. These genes encode members of the fibroblast growth factor (FGF) [28] and the TGFβ [29] gene families, their receptors, epidermal growth factor-receptor (EGF-R) [30], insulin-like growth factor I (IGF-I) [31] and members of the surfactant-associated protein family [32].

**Table 3:** Several genes involved in lung development and expressed with no sex difference

**FGF signaling pathway**		**TGF-beta signaling pathway**	**Surfactant associated proteins**	**Others**
				
Spry	Fgfbp1	Tgfb1	Sftpa	Egfr
Fgf1	Fgfr1op2	Tgfb2	Sftpb	Igf1
Fgf7	Fgfrap1	Tgfb3	Sftpc	
Fgf13	Akr1b8	Tgfbr1	Sftpd	
Fgf18	Fgfrl1	Tgfbr2		
Fgfr1		Tgfbr3		
Fgfr2				
Fgfr3				
Fgfr4				

### Genes with sexually dimorphic expression

**Figure 2:**
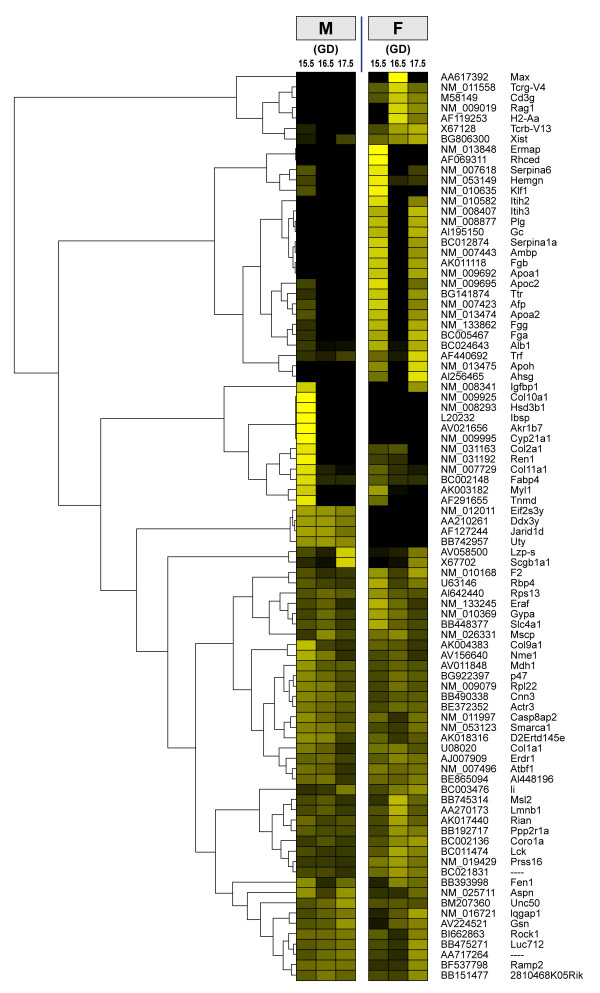
**Hierarchical clustering and expression profile of differentially expressed genes **Hierarchical clustering and expression profile of genes presented in Table 2. The day of gestation is indicated at top. Accession numbers and gene symbols are presented. For each gene individually, each value was normalized by division with the root mean square calculated from the six values. As a consequence, the relative values obtained for one gene cannot be compared to those of other genes. Expression levels are presented from black (no expression) to yellow (high relative expression level). The clustering was generated using the Genesis 1.6.0 Beta1 software (agglomeration rule of average linkage and Euclidean measurement distance) [26]. M, male; F, female.

Genes with sexually dimorphic expression in mouse lungs on GDs 15.5, 16.5, and 17.5 are presented in Table 2. These genes are distributed according to an adaptation of the functional categories defined by the Gene Ontology Consortium [33]. Only 33 genes displayed a sex difference on GD 16.5 compared with 62 and 38 on GD 15.5 and 17.5, respectively. Of these, similar numbers of genes showed higher expression levels for each sex on GD 15.5, while 21 of the 33 genes identified on GD 16.5, and 32 of the 38 genes identified on GD 17.5 presented higher expression in the female lung. Hierarchical clustering and expression profile of these genes are presented in Figure 2.

## Discussion

This study targeted elucidation of a sex difference in the fetal lung during a three-day gestation period overlapping PTII cell maturation. This sex difference results in more concern for a poorer prognosis for respiratory distress in premature male infants. The genes identified here, with a sexually dimorphic expression during the gestation period examined in the mouse, likely contain key genes involved in PTII cell maturation. Further studies are essential to identify and to characterize these key genes, as our data indicate that PTII cell maturation is not the only aspect of lung development affected by sex during this period of gestation.

**Figure 3:**
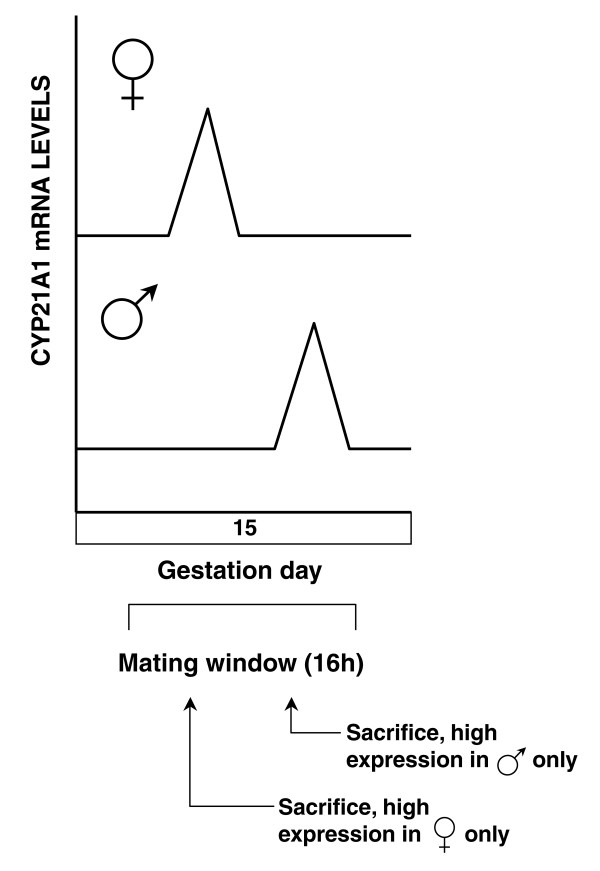
**Hypothesis concerning the expression profile ofsome differentially expressed genes **Our previous results suggest that Cyp21a1 is expressed at different precise gestation time on GD 15 for each gender [27] as shown in the present figure. This hypothesis would explain why the expression profile of Cyp21a1 on GD15.5 varied from litter to litter and presented one of the following patterns : 1) high expression in female fetal lungs only; 2) high expression in male fetal lungs only; or 3) no elevated expression in male and female fetal lungs. Because Balb/C is an inbred strain, the mating window of 16h would explain the variation between litters. Genes presenting such an expression profile cannot be studied by microarrays from a pool of many litters because the sex difference in expression could be lost.

Analysis of the data has to be performed in the context of lung development where a transient delay in the surge of surfactant synthesis is observed. Therefore, as in the case of the surge of surfactant synthesis, some genes can be subject to a transient delay in expression for one sex. The case of the Cyp21a1 gene clearly illustrates this occurrence. Recently, we showed that Cyp21a1, a gene involved in corticosteroid synthesis, is expressed specifically on GD 15 in the fetal mouse lung [27]. Of the six litters studied on GD 15.5 and obtained using the same mating window of ± 8 hours, two litters presented high expression of Cyp21a1 in females only, whereas one litter had high expression of this gene only in male fetuses. The three remaining litters did not show any elevated levels of expression for this gene (Figure 3). The exact gestation time at which the pregnant females were sacrificed varied from litter to litter according to the time where the females were mated during the mating window of ± 8h. Knowing that Balb/C is an inbred strain, variations from litter to litter should represent the expression pattern of Cyp21a1 gene at different times on GD 15. Consequently, our results strongly suggested that Cyp21a1 should present a narrow peak of expression on GD 15 with a delay for one sex [27]. In the present study, expression of Cyp21a1 (Id. #30) was detected by gene profiling only in males on GD 15.5. Real-time PCR analysis of the two litters pooled to prepare probes on GD15.5 revealed that Cyp21a1 was expressed at high levels only in male lungs (one out of two litters) (data not shown). Therefore, our microarray results are compatible with our previous report [27]. In this example, the fact that we detected expression only in male fetuses by microarrays depended only on the litters used. Therefore, for the analysis of our microarray results, the identity of the sex where expression is higher compared to the other sex has to be considered with caution. Other animals, mated some hours before or after the pregnant females used in this study within the same mating window, could show elevated expression levels for the other sex, or even present no sex difference at all, as a consequence of a delayed expression in one sex.

Recently, we reported that all the genes involved in corticosteroid synthesis from cholesterol in the mature adrenal gland are transiently expressed in the developing mouse lung on GD 15 [27]. Hsd3b1 (Id. #28) encodes the 3β-hydroxysteroid dehydrogenase type 1 enzyme that is involved in this cascade of reactions (pregnenolone → progesterone) along with Cyp21a1 (Id. #30) (progesterone → deoxycorticosterone) as mentioned already. Both genes were also found by microarray analysis to be expressed transiently on GD 15 with a sex difference.

Some other genes related to steroid hormone metabolism/regulation are expressed also with a sex difference in fetal mouse lungs. This is the case for Akr1b7 (Id. #29) which showed a sex difference in expression on GD 15.5. This gene encodes an aldose reductase-like protein whose major function is detoxification of isocaproaldehyde generated by conversion of cholesterol to pregnenolone by the enzyme P450scc [34], the latter also being expressed on GD 15.5 in the mouse fetal lung [27]. Scgb1a1 gene (Id. #53) is expressed also on GD 15.5 with a sex difference. This gene encodes the secretoglobin family 1 A member 1, also called Clara cell secretory protein (CCSP), which is required for the appearance of Clara cells secretory granules and thus participates to the composition of the epithelial lining fluid [35]. Scgb1a1 gene can be induced by glucocorticoids [36] and CCSP can act as an anti-inflammatory protein [37]. It was also shown to bind progesterone [38]. Some other genes were not detected by microarrays although they are known to be expressed with a sex difference in lung development as evidenced by real-time PCR. These genes include 17β-hydroxysteroid dehydrogenase (HSD) type 2 and type 5 [18] which are expressed at low levels in the lung, probably below the minimal threshold of sensitivity of the microarray technology.

Some genes involved in the metabolism of non-steroidal hormones were also found to be expressed differentially between genders. This is the case for Ttr (Id. #32), which codes for transthyretin, a common plasma carrier protein for thyroid hormones and vitamin A metabolites [39]. Interestingly, retinoids are important regulators of normal epithelial cell differentiation and proliferation [40] and are involved in lung development [41]. Ren1 (Id. #33) encodes for renin 1 and presented a sex difference in expression on GD 15.5. Genes of the renin-angiotensin system are known to be expressed in the fetal lung and, interestingly, angiotensin II can present mitogenic effects on human lung fibroblasts through the activation of the type 1 angiotensin II receptor [42]. In our study, Ace (angiotensin II converting enzyme), Agtr1, and Agtr2 (angiotensin II receptor type 1 and type 2) are expressed with no sex difference (data not shown).

Our results demonstrate that genes coding for apolipoprotein (apo) AI (Id. #45), apoAII (Id. #47), apoCII (Id. #46), and apoH (Id. #44) are co-expressed in the developing lung. To our knowledge, none of these genes has been found expressed in the lung. All of these genes present a sex difference in favor of females in the developing lung on GD 15.5 and 17.5, with no detectable expression on GD 16.5. As demonstrated by real-time PCR, 17βHSD types 2 and 5 were also expressed on GDs 15.5 and 17.5 with a sex difference for the majority of litters, but not on GD16.5 [18]. Our results suggest that the expression of these four apolipoprotein genes in the developing lung is under active modulation. 17β-HSD types 5 and 2 genes are involved in androgen synthesis and inactivation, respectively. Such a similar pattern of expression for these apolipoproteins and 17β-HSD genes may suggest a common regulatory mechanism, or an effect of androgens on expression of these apolipoprotein genes. Apolipoproteins are constituents of circulating lipoproteins. ApoCII is an essential cofactor/activator of lipoprotein lipase (LPL) [43,44] while apoH was shown to enhance LPL [45]. This enzyme catalyses the hydrolysis of the triacylglycerol component of circulating lipoproteins (chylomicrons and very low density lipoproteins (VLDLs)). It was reported that the presence and activity of LPL in the lung may be important for surfactant production [46]. LPL expression was detected with no sex difference. ApoAI [47] and apoAII [48] are major protein components of high density lipoproteins (HDLs). ApoAI is a potent activator of lecithin:cholesterol acyltransferase (LCAT), an HDL-associated enzyme playing a role in reverse cholesterol transport. It is possible that the sex difference in expression of four apolipoprotein genes in the lung could explain in part the sex difference in surfactant lipids observed on GD 17.5; although such a role in the surge of surfactant synthesis has never been suspected for these genes before the present study. It is also noteworthy that many genes involved in lipid metabolism were expressed with no sex difference during each gestational day studied (data not shown). For example, this is the case for genes encoding for LDL receptor, LDL receptor-related protein 1, phospholipid transfer protein, and HMG-CoA-reductase (3-hydroxy-3-methylglutaryl-Coenzyme A reductase), the latter being known to catalyze the rate-limiting step of cholesterol synthesis.

Along with other pathways, fibroblast growth factors (FGFs) and TGFβ signaling pathways have been shown to be involved in many aspects of lung development. We observed expression of many genes that belong to these pathways (Table 3). However, in the present study, these genes exhibit no sexually dimorphic expression. This is in line with our previous study by real-time PCR that showed expression of 5 genes involved in lung maturation on GDs 16.5 and 17.5 with no sex difference, namely, LIF, IGF-I, KGF (FGF-7), EGF-R, and neuregulin-1 [18]. Although it is possible that these genes could be expressed with a sex difference within a time window not covered by our studies, it seems that the sex difference in PTII cell maturation could rely on other genes.

Tissue remodeling and structure also seem subject to sexual differences in gene expression. Indeed, two genes involved in apoptosis, namely, Rock1 (Rho-associated coiled-coil forming kinase 1) (Id. #1) and Casp8ap2 (caspase 8 associated protein 2) (Id. #2) are differentially expressed on GD 16.5 between genders, while many genes involved in cell adhesion are expressed with a sex difference on GD 15.5. In addition, IGF signaling pathway has been shown to be important in mouse lung development where it is involved in regulation of cell proliferation and differentiation [31,49,50]. Igfbp1 (Id. #9) presented a sex difference in expression on GD 15.5, whereas Igfbp1 deficiency was associated with massive hepatocyte apoptosis [51].

In preparation for important immunological challenges related to its function, the developing lung acquires many elements associated with innate and adaptive immunity. Our data suggest that modulation of several genes involved in immune functions of the lung is subject to sex differences. Indeed, the predominating functional category of genes showing sexual dimorphism on GD 16.5 concerns "immune functions". It has been shown that many signaling pathways involved in lung morphogenesis and immune responses are crosslinked [52]. These include TTF-1, GATA6 and HNF-3β transcription factors; and FGF- and NF-κ B-dependant signaling pathways. However, we did not detect any sex difference in expression for these genes.

## Conclusion

This study revealed that many genes are expressed with a gender difference in the fetal lung. Although we focused on a brief gestational period overlapping the surge of surfactant synthesis, our data demonstrates that PTII cell maturation is not the only aspect of lung development under the influence of fetal sex. We suggest that, among the genes identified here, some are related to the transient delay in lung maturation observed for males. This would help identify novel genes involved in the physiopathology of respiratory distress of the neonate.

## Competing interests

The author(s) declare that they have no competing interests.

## Authors' contributions

MS participated in the design of the study, performed the data analysis and wrote the manuscript. PRP participated in the design of the study and helped to draft the manuscript. YT conceived the study, participated in its design and in its coordination. All authors read and approved the final manuscript.
